# Low Hypoperfusion Intensity Ratio Is Associated with a Favorable Outcome Even in Large Ischemic Core and Delayed Recanalization Time

**DOI:** 10.3390/jcm10091869

**Published:** 2021-04-26

**Authors:** Jang-Hyun Baek, Young Dae Kim, Ki Jeong Lee, Jin Kyo Choi, Minyoul Baik, Byung Moon Kim, Dong Joon Kim, Ji Hoe Heo, Hyo Suk Nam

**Affiliations:** 1 Department of Neurology, Kangbuk Samsung Hospital, Sungkyunkwan University School of Medicine, Seoul 10415, Korea; janghyun.baek@gmail.com; 2 Department of Neurology, Severance Hospital, Yonsei University College of Medicine, Seoul 03722, Korea; neuro05@yuhs.ac (Y.D.K.); juno8263@gmail.com (K.J.L.); jksnail85@yuhs.ac (J.K.C.); minyoulbaik@yuhs.ac (M.B.); jhheo@yuhs.ac (J.H.H.); 3 Integrative Research Institute for Cerebral and Cardiovascular Diseases, Yonsei University College of Medicine, Seoul 03722, Korea; 4 Department of Neurology, National Health Insurance Service Ilsan Hospital, Ilsan 10444, Korea; 5 Department of Neurology, Seoul Medical Center, Seoul 02053, Korea; 6 Department of Radiology, Severance Hospital, Yonsei University College of Medicine, Seoul 03722, Korea; bmoon21@hanmail.net (B.M.K.); djkimmd@yuhs.ac (D.J.K.)

**Keywords:** hypoperfusion, collaterality, stroke, outcome, thrombectomy

## Abstract

In ischemic brain tissue, hypoperfusion severity can be assessed using the hypoperfusion intensity ratio (HIR). We evaluated the link between HIR and clinical outcomes after successful recanalization by endovascular treatment. We retrospectively reviewed 162 consecutive patients who underwent endovascular treatment for intracranial large vessel occlusion. The HIR was calculated using an automated software program, with initial computed tomography perfusion images. The HIR was compared between patients with and without favorable outcomes. To observe the modifying effect of the HIR on the well-known major outcome determinants, regression analyses were performed in the low and high HIR groups. The median HIR value was significantly lower in patients with a favorable outcome, with an optimal cut-off point of 0.54. The HIR was an independent factor for a favorable outcome in a specific multivariable model and was significantly correlated with the Alberta Stroke Program Early Computed Tomography Score (ASPECTS). In contrast to the high HIR group, the low HIR group showed that ASPECTS and onset-to-recanalization time were not independently associated with a favorable outcome. Finally, the low HIR group had a more favorable outcome even in cases with an unfavorable ASPECTS and onset-to-recanalization time. The HIR could be useful in predicting outcomes after successful recanalization.

## 1. Introduction

Hypoperfusion severity and duration are important factors affecting the clinical outcome of patients with acute ischemic stroke who undergo endovascular treatment (EVT). Collateral status is a commonly used method that reflects hypoperfusion severity. Robust collateral flow is associated with smaller ischemic core lesions and slower progression, which may lead to improved clinical outcomes [1,2,3,4]. Moreover, time windows for EVT eligibility can be determined based on the collateral status. Patients with better collateral flow may have a more favorable outcome, even in cases in which recanalization is delayed [5].

Hypoperfusion severity can be assessed directly using the hypoperfusion intensity ratio (HIR) [6]. The HIR reflects the proportion of the critically hypoperfused lesion (T_max_ > 10 s) in the whole hypoperfused lesion (e.g., T_max_ > 6 s) on perfusion images [7,8]. The HIR correlates well with the quality of the collateral status and is considered a quantitative marker of the collateral status [7,9,10]. Furthermore, the HIR has been reported to be significantly associated with initial stroke severity, target mismatch profile, and infarct growth [6,7,8,11]. Based on the collateral nature of the HIR, we assumed that the clinical outcome after EVT may be affected by the HIR, as in the case of the collateral status.

Accordingly, we hypothesized that a low HIR is associated with a favorable outcome, even in patients with a lower Alberta Stroke Program Early Computed Tomography Score (ASPECTS) or longer onset-to-recanalization time in patients who achieved recanalization through EVT.

## 2. Materials and Methods

### 2.1. Participants

We retrospectively reviewed consecutive patients with acute stroke who underwent EVT for intracranial large artery occlusion in the anterior circulation between January 2016 and April 2020. All patients had been registered to a prospectively maintained registry of a tertiary stroke center. EVT was considered for patients who met the following criteria: (1) Computed tomography (CT) angiography-confirmed, endovascularly accessible intracranial occlusions associated with neurological symptoms; (2) in the earlier study period, within 8 h from stroke onset; patients within 8–12 h were also considered if they had an ASPECTS ≥ 7; (3) the eligibility criteria of the Diffusion and Perfusion Imaging Evaluation for Understanding Stroke Evolution 3 (DEFUSE 3) and DWI or CTP Assessment with Clinical Mismatch in the Triage of Wake-Up and Late Presenting Strokes Undergoing Neurointervention with Trevo (DAWN) trials were applied to patients within 6–24 h from stroke onset; and (4) initial National Institutes of Health Stroke Scale (NIHSS) score ≥ 4. We also preferably performed EVT for patients with a premorbid modified Rankin Scale (mRS) score ≤ 3. Patients eligible for intravenous tissue plasminogen activator treatment were treated with 0.9 mg/kg tissue plasminogen activator. EVT procedures were performed under local anesthesia. In most cases, a stent retriever was used as the front-line EVT modality and a balloon-guiding catheter was routinely used. The detailed procedure is described elsewhere [12].

We included patients with intracranial large vessel occlusion, which was defined as occlusion of the intracranial internal carotid artery or middle cerebral artery M1 or proximal M2 segment, and those in whom the occlusion was successfully recanalized by EVT. Successful recanalization was defined as modified Thrombolysis in Cerebral Infarction grade 2b or 3.

### 2.2. Imaging and Clinical Data

All patients underwent CT as soon as they arrived at the hospital. The pretreatment CT scan included non-contrast CT images, CT angiography, and CT perfusion images. To obtain the HIR, hypoperfusion lesion volumes were quantitatively assessed from CT perfusion images using an automated software (RAPID, iSchemaView, Menlo Park, CA, USA). The lesion volume was measured by a stroke neurologist without any manual correction. The HIR was calculated as the ratio of the lesion volume with T_max_ > 10 s to T_max_ > 6 s [7].

Data on all variables used in this study were collected from the prospective registry of patients with acute stroke. The functional outcome was assessed based on the mRS score at 3 months after stroke onset and was primarily evaluated by stroke neurologists during the patient’s routine clinical follow-up at 3 months ± 2 weeks. If a patient was unable to present to the follow-up appointment, a stroke neurologist or a trained nurse interviewed the patient or their family via telephone to determine the mRS score using a standard questionnaire. A favorable outcome was defined as an mRS score of 0–2. Intracerebral hemorrhage (ICH) was assessed on follow-up CT or magnetic resonance images obtained 24 ± 6 h after EVT. The assessment of ICH was based on the consensus of stroke neurologists, neurointerventionalists, and neuroradiologists during the regular stroke conference. The determination of ICH was immediately entered into the prospective registry. ICH was regarded as symptomatic if the patient’s NIHSS score increased by ≥ 4 according to the European Cooperative Acute Stroke Study III [13].

The ASPECTS was reassessed by two independent neurointerventionalists and stroke neurologists [14], who only had access to the initial non-contrast CT images and who were completely blinded to any endovascular outcome and clinical information, except for the lesion side. The interrater agreement for the ASPECTS was good (intraclass correlation coefficient, 0.657). Discrepancies in the assessment of cases were resolved by consensus.

### 2.3. Statistical Analysis

Based on the study hypotheses, we performed the following analyses step by step. First, to evaluate the association between the HIR and clinical outcomes, patients were assigned to the favorable outcome group or the unfavorable outcome group. Then, (1) HIR values were compared among groups along with patient demographics, risk factors for stroke, clinical and radiological severity of stroke, and symptomatic ICH. Student’s *t*-test, the Mann–Whitney *U* test, the chi-square test, and Fisher’s exact test were used for group comparisons. (2) We calculated the optimal cut-off point of the HIR for a favorable outcome using the Youden index. Through the receiver operating characteristic curve analysis, the area under the curve was also calculated. (3) To quantify the association between the HIR and a favorable outcome, we performed univariable binary logistic regression analyses for a favorable outcome. Multivariable analysis was also performed to identify whether the HIR was an independent variable for a favorable outcome. Variables with a *p*-value of < 0.1 in univariable analyses were entered in the multivariable model.

Second, to evaluate whether the HIR can modify the effect of the well-established outcome determinants (the ASPECTS and onset-to-recanalization time) on clinical outcomes, the HIR was dichotomized into low and high by its optimal cut-off point for a favorable outcome. According to the low HIR group and the high HIR group, the association between the ASPECTS and a favorable outcome was analyzed by logistic regression analyses. The association between onset-to-recanalization time and a favorable outcome was also analyzed in the same way. We also plotted regression curves by combining the HIR, the ASPECTS, onset-to-recanalization time, and a favorable outcome. For this, patients were assigned to one of the four groups according to the HIR and ASPECTS: (1) the low HIR and small core (ASPECTS ≥ 8) group, (2) the low HIR and large core (ASPECTS < 8) group, (3) high HIR and small core group, and (4) the high HIR and large core group. Regression curves of onset-to-recanalization time for a favorable outcome in the respective groups were compared.

A *p*-value of < 0.05 was considered statistically significant for 95% confidence intervals (CIs). All statistical analyses were performed using R software (version 4.0.1, The R Foundation, r-project.org, Vienna, Austria).

## 3. Results

**Figure 1:**
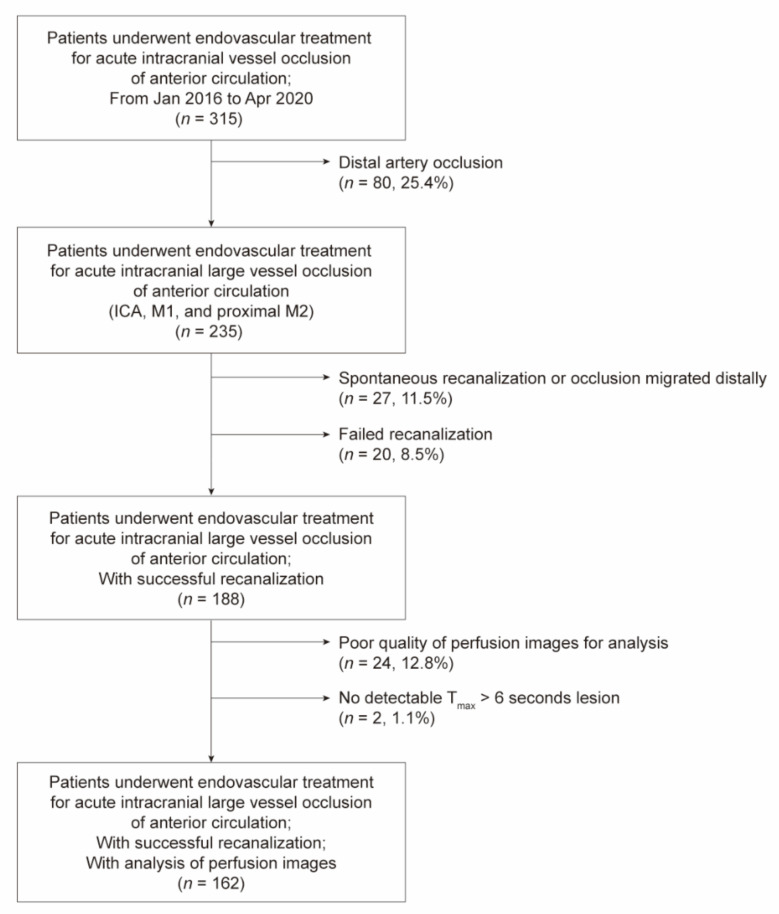
Patients selection flow chart.

Of the 188 patients who underwent successful recanalization of intracranial large vessel occlusion, perfusion images of 26 (13.9%) were not analyzable due to poor quality, including motion artifacts (*n* = 24) and undetectable T_max_ > 6 s perfusion lesions (*n* = 2). A total of 162 patients (mean age, 70.7 ± 12.8 years; male, 51.9%) who met the inclusion criteria were analyzed (Figure 1). The median imaging-to-recanalization time was 126.0 min (interquartile range [IQR], 97.0–153.5). The mean lesion volumes of T_max_ > 6 s and T_max_ > 10 s were 160.8 mL (IQR, 104.2–205.2) and 80.3 mL (IQR, 30.3–136.0), respectively. The median HIR value was 0.51 (IQR, 0.29–0.68).

### 3.1. Association between the HIR and Clinical Outcomes

Of the patients included, 85 (52.5%) patients had a favorable outcome. The median HIR value was significantly lower in patients with a favorable outcome than those with an unfavorable outcome (0.45 [IQR, 0.15–0.54] vs. 0.60 [IQR, 0.44–0.73]; *p* < 0.001; Table 1). The optimal cut-off point of the HIR for a favorable outcome was 0.54 (sensitivity, 63.6%; specificity, 74.1%). The area under the curve of the HIR to predict a favorable outcome was 0.728 (95% CI, 0.651–0.805; *p* < 0.001).

**Figure 2:**
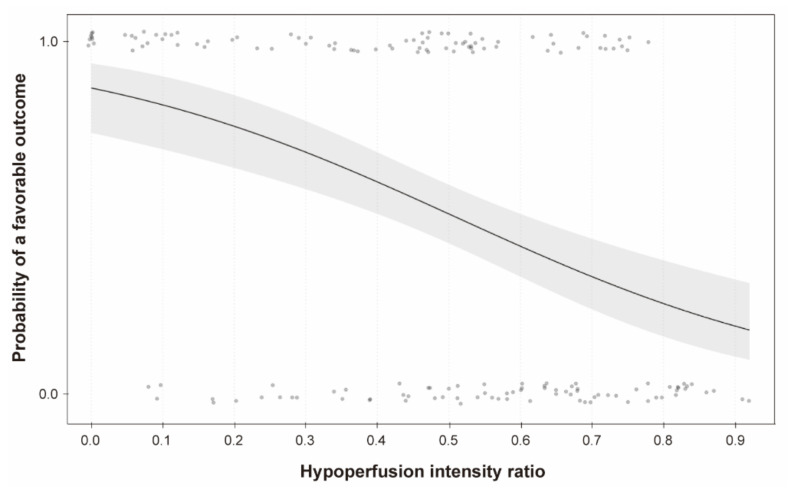
Association between the hypoperfusion intensity ratio and a favorable outcome.

**Figure 3:**
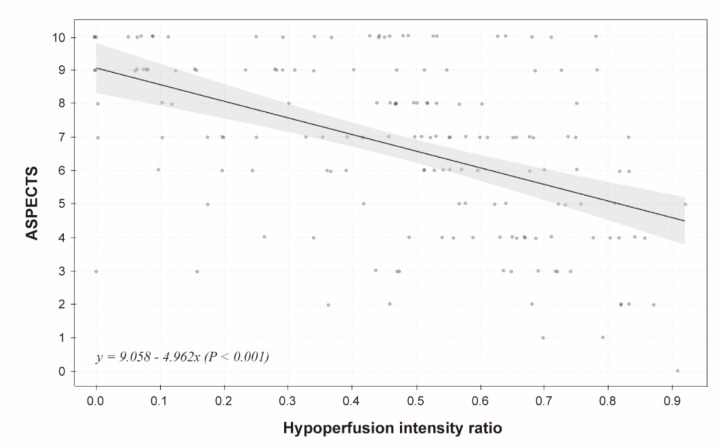
Association between hypoperfusion intensity ratio and Alberta Stroke Program Early CT Score (ASPECTS).

In univariable logistic regression analysis, the HIR was significantly associated with a favorable outcome (odds ratio [OR], 0.69 per 0.1; 95% CI, 0.59–0.81; *p* < 0.001; Figure 2), although it was not an independent factor for a favorable outcome after adjustment (adjusted OR [aOR], 0.84 per 0.1; 95% CI, 0.68–1.03; *p* = 0.094; Model 1 in Table 2). We found that the HIR was significantly correlated with the ASPECTS (correlation coefficient, −0.49; *p* < 0.001; Figure 3). Considering the collinearity, the HIR was independently associated with a favorable outcome in a model without the ASPECTS (aOR, 0.76 per 0.1; 95% CI, 0.62–0.92; *p* = 0.006; Model 2 in Table 2).

### 3.2. Association between the ASPECTS, Onset-To-Recanalization Time, and Clinical Outcomes According to the HIR

**Figure 4:**
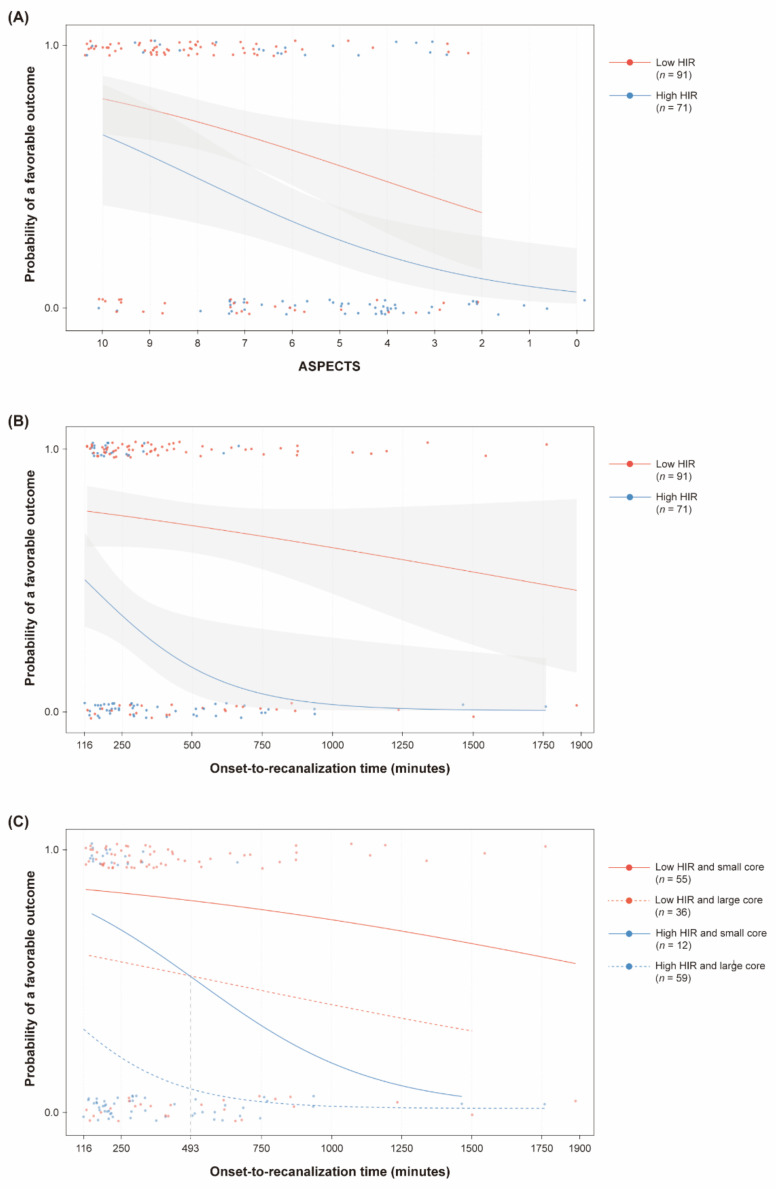
Influence of the Alberta Stroke Program Early CT Score (ASPECTS) and onset-to-recanalization time on clinical outcomes based on the hypoperfusion intensity ratio (HIR). (**A**) Although the ASPECTS was not significantly associated with a favorable outcome in the low HIR group (*p* = 0.178), it was an independent factor for a favorable outcome in the high HIR group (*p* = 0.012). (**B**) For patients with a high HIR, the chances of a favorable outcome sharply decrease when the onset-to-recanalization time is relatively short. (**C**) Considering both the HIR and ASPECTS, patients with a low HIR (<0.54) and small core (ASPECTS ≥ 8) have the best clinical outcome in all ranges of onset-to-recanalization time (*p* < 0.05). In contrast, patients with a high HIR (≥ 0.54) and large core (ASPECTS < 8) have the worst outcome in all ranges of onset-to-recanalization time. Patients with a high HIR and small core have a more favorable outcome than those with a low HIR and large core when onset-to-recanalization time is relatively short. However, for patients with a high HIR and small core, the chance of a favorable outcome decreases more drastically with the course of time and then finally reverses from a particular point of onset-to-recanalization time.

**Figure 5:**
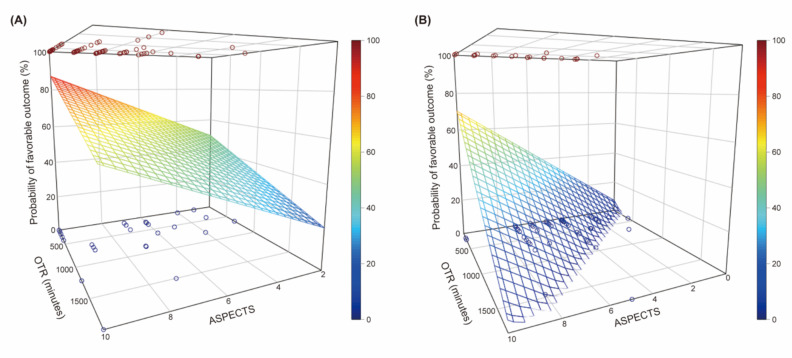
The probability of a favorable outcome according to Alberta Stroke Program Early CT Score (ASPECTS) and onset-to-recanalization time in the low hypoperfusion intensity ratio (HIR) group (**A**) and the high HIR group (**B**). (**A**) In the low HIR group, the probability of a favorable outcome was still above 20% even under the lower ASPECTS and longer onset-to-recanalization time. (**B**) The probability of a favorable outcome was sharply decreased as the ASPECTS and onset-to-recanalization time changed in the high HIR group.

Based on the optimal cut-off point of the HIR for a favorable outcome, 91 (56.2%) patients were assigned to the low HIR group (HIR < 0.54). The effect of the ASPECTS and onset-to-recanalization time on a favorable outcome was different between the low and high HIR groups. In the low HIR group, only the initial NIHSS score was associated with a favorable outcome. The ASPECTS (aOR, 1.20; 95% CI, 0.92–1.56; *p* = 0.178) and onset-to-recanalization time (aOR, 0.97 per 30 min; 95% CI, 0.94–1.01; *p* = 0.194) were not significantly associated with a favorable outcome (Table 3; Figure 4A,B). In contrast, in the high HIR group, the ASPECTS was an independent factor for a favorable outcome (aOR, 1.49; 95% CI, 1.09–2.03; *p* = 0.012). Although onset-to-recanalization time was not independently associated with a favorable outcome (OR, 0.92 per 30 min; 95% CI, 0.79–1.07; *p* = 0.283) in the high HIR group, the probability of a favorable outcome decreased sharply when onset-to-recanalization time was relatively short (Figure 4B). As a whole, 3-dimensional regression planes showed that the probability of a favorable outcome was still above 20% even under the lower ASPECTS and longer onset-to-recanalization time in the low HIR group (Figure 5). In contrast, the combined probability of a favorable outcome was sharply decreased as the ASPECTS and onset-to-recanalization time changed in the high HIR group.

Based on the HIR and ASPECTS, 55 (34.0%) patients had a low HIR and small core, 12 (7.4%) patients had a high HIR and small core, 36 (22.2%) patients had a low HIR and large core, and 59 (36.4%) patients had a high HIR and large core. The decreasing trend of a favorable outcome was significantly different between the groups (*p* < 0.05). Patients with a low HIR and small core had the highest probability of a favorable outcome throughout the study period, while patients with a high HIR and large core had the lowest probability. Patients with a high HIR and small core had a more favorable outcome than those with a low HIR and large core when the onset-to-recanalization time was relatively short. However, their clinical outcomes were reversed after the onset-to-recanalization time of 493 min since the probability of a favorable outcome decreased substantially in patients with a high HIR and small core (Figure 4C).

## 4. Discussion

In this study, we found that clinical outcomes were substantially different based on the HIR value. A low HIR led to a favorable outcome even in cases with a low ASPECTS and longer onset-to-recanalization time. In contrast, in patients with a high HIR, the ASPECTS was still important for clinical outcome. In addition, the chance of a favorable outcome drastically decreased when the onset-to-recanalization time was relatively short in the high HIR group. Although a study simply reported the association between the HIR and the clinical outcome [7], our study showed more specifically that the probability of a favorable outcome based on the onset-to-recanalization time and the ASPECTS was clearly disparate according to the HIR. The HIR might be an ancillary preprocedural marker to predict the clinical outcomes after EVT.

The collateral nature of HIR was more clearly observed in patients with a high HIR and small core. They had a more favorable outcome than patients with a large core in the shorter onset-to-recanalization time, where the clinical outcome was mainly determined by their core size. However, in the high HIR group, clinical outcome was less favorable despite a small core. Patients with good collaterals could have a favorable outcome even with a longer onset-to-recanalization time; however, the probability of a favorable outcome was substantially reduced in patients with poor collaterals [5]. In addition, the effect of rapid recanalization on a favorable outcome was deliberated in patients with good collaterals, similar to that seen in the low HIR group in our study. In the same manner, it appears that a low HIR may also have a favorable effect on infarct development and its progression. In a previous study, a high HIR was significantly associated with greater infarct growth [7,15,16,17]. Our study showed that the HIR alone was an independent factor for a favorable outcome in the multivariable model without considering the ASPECTS. After adjusting the ASPECTS, the HIR was not an independent factor for a favorable outcome, which would be mainly because of tight correlation between the HIR and the ASPECTS.

Specific perfusion deficit volumes—for examples, ischemic core volume (relative cerebral blood flow < 30%) and penumbral volume (T_max_ > 6 s) can be associated with clinical outcome. In fact, also in this study, ischemic core volume was independently associated with a favorable outcome, as ASPECTS does. Although such a perfusion parameter is still important in determining clinical outcome, HIR seems still valuable in clinical practice. Along with the common perfusion deficit parameters, HIR could be one of the influencing factors for clinical outcome. Based on its collateral nature, HIR might differentially affect clinical outcome even under the similar ischemic core status or onset-to-recanalization time.

Calculation of the HIR can be clinically advantageous compared to the evaluation of the collateral status, a typical marker reflecting hypoperfusion severity. First, although the assessment of collateral status is easy to perform on pretreatment CT angiography images, quantifying the collateral status are inconsistent and may be qualitative or merely categorical. Most importantly, the evaluation of the collateral status is subjective and reviewer dependent. In contrast, the HIR is more objective in nature. The use of an automated software for the calculation of the HIR can minimize the potential interrater variability and is associated with greater generalization capacity. In addition, the HIR is essentially quantitative as a continuous value, which enables quantitative analysis and can reveal subtle differences in hypoperfusion severity. Furthermore, the HIR can be assessed even in a magnetic resonance-based eligibility strategy. Typical CT angiography-based collateral assessment methods cannot be applied to time-of-flight magnetic resonance angiography. However, the HIR can be calculated with perfusion-weighted images taken using the magnetic resonance technique; thus, its use may be more generalizable. Second, based on the specific cut-off point for a favorable outcome, the HIR may constitute a clinical element that can be used to determine EVT eligibility. In a retrospective study, patients eligible for EVT had a more favorable HIR value than those ineligible for EVT [18]. The HIR may also be a dependable eligibility factor in interfacility transfer since the HIR can significantly predict future infarct growth [8]. According to the recent guidelines for EVT, perfusion imaging with lesion volume analysis has been widely performed to determine the EVT eligibility. Unlike the earlier situation where simple neuroimaging is favored, advanced neuroimaging, including software-based lesion volume analysis, has been increasingly used for EVT eligibility since the DEFUSE 3 and DAWN trials. Although lesion volume analysis is not available in all stroke centers, it is becoming increasingly popular.

In previous studies, a cut-off point of the HIR to predict a favorable functional outcome were not calculated; the cut-off points of the HIR for the collateral status and infarct growth were calculated as 0.40 and 0.50, respectively [7,8,9]. Most studies merely dichotomized the HIR by its median value, with a range of 0.30–0.45, to evaluate the association of the dichotomized HIR value with the fluid-attenuated inversion recovery (FLAIR) hyperintense vessel sign, infarct growth, or first-pass effect in mechanical thrombectomy [7,19,20,21]. In these studies, a median HIR value of <0.40 was significantly associated with a positive outcome [7]. In our study, the cut-off point of the HIR for a favorable outcome was calculated as 0.54, which was higher than the 0.40 value, although direct comparison is limited. This may be due to the fact that our study included only patients who underwent successful recanalization. Successful recanalization may give a more favorable clinical outcome in patients with higher HIR values than those with lower HIR values.

This study has several limitations. First, this study was performed retrospectively using prospectively collected data on consecutive patients diagnosed with acute ischemic stroke. The treatment eligibility criteria and protocol were revised during the study period according to the guidelines. Furthermore, the study results from a single center can limit generalizability. Although the HIR value was a rather objective finding obtained from an automated software, the cut-off point of the HIR for a favorable outcome and its significance may be limited to the specific study population. Thus, the results of this study should be interpreted with caution. Second, we evaluated the significance of the HIR only in patients who underwent successful recanalization; however, considering its collateral nature, the HIR may also influence the clinical outcomes in cases of EVT failure, because good collaterals were significantly associated with a favorable outcome even for patients in whom recanalization was unsuccessful [22]. Further studies are needed to demonstrate that the HIR could be a marker in case of EVT failure and to validate the current cut-off in that group.

## 5. Conclusions

The HIR was associated with clinical outcomes in patients who underwent successful recanalization using EVT. Specifically, a low HIR was associated with a favorable outcome. EVT might need to be considered for patients with a low HIR despite the relatively unfavorable ischemic core status and time profile. Further prospective studies are needed to establish the EVT eligibility criteria based on HIR.

## Figures and Tables

**Table 1:** Comparison of clinical variables and hypoperfusion intensity ratio between patients with and without favorable outcome.

Variables	All Patients (*n* = 162)	Favorable Outcome (*n* = 85)	Unfavorable Outcome (*n* = 77)	*p*-Value
Age	70.7 (± 12.8)	67.6 (± 12.9)	74.1 (± 11.8)	0.001
Sex, male	84 (51.9)	48 (56.5)	36 (46.8)	0.216
Hypertension	122 (75.3)	65 (76.5)	57 (74.0)	0.719
Diabetes	75 (46.3)	31 (36.5)	44 (57.1)	0.008
Hypercholesterolemia	77 (47.5)	39 (45.9)	38 (49.4)	0.659
Smoking	31 (19.1)	21 (24.7)	10 (13.0)	0.058
Coronary artery disease	29 (17.9)	19 (22.4)	11 (14.3)	0.187
Atrial fibrillation	80 (49.4)	38 (44.7)	42 (54.5)	0.211
Occlusion site				0.406
Internal carotid artery	64 (39.5)	31 (36.5)	33 (42.9)	
Middle cerebral artery	98 (60.5)	54 (63.5)	44 (57.1)	
Initial NIHSS score	15.0 [11.0; 19.8]	12.0 [7.0; 16.0]	19.0 [15.0; 20.0]	<0.001
Use of intravenous tPA	78 (48.1)	50 (58.8)	28 (36.4)	0.004
ASPECTS	7.0 [5.0; 9.0]	8.0 [7.0; 9.0]	6.0 [4.0; 7.0]	<0.001
Onset-to-recanalization, min	298.5 [197.0; 583.5]	274.0 [190.0; 434.0]	336.0 [212.0; 674.0]	0.045
Symptomatic ICH	10 (6.2)	4 (4.7)	6 (7.8)	0.520
Volume of rCBF < 30% (mm^3^)	33.7 (± 53.6)	12.0 (± 21.7)	57.6 (± 66.7)	<0.001
Volume of T_max_ > 6 s (mm^3^)	160.8 (± 76.7)	140.4 (± 67.6)	183.2 (± 80.2)	<0.001
Hypoperfusion intensity ratio	0.51 [0.29; 0.68]	0.45 [0.15; 0.54]	0.60 [0.44; 0.73]	<0.001

Values in parentheses represent the standard deviation (±) or number of patients (%); brackets represent first and third quartiles, respectively. NIHSS, National Institutes of Health Stroke Scale; tPA, tissue plasminogen activator; ASPECTS, Alberta Stroke Program Early Computed Tomography Score; ICH, intracerebral hemorrhage; rCBF, relative cerebral blood flow.

**Table 2:** Multivariable analyses for a favorable outcome.

Variables	Model 1	*p*-Value	Model 2	*p-*Value
	aOR (95% CI)		aOR (95% CI)	
Age	0.97 (0.94–1.01)	0.153	0.98 (0.94–1.01)	0.171
Diabetes	0.95 (0.40–2.25)	0.899	0.77 (0.34–1.76)	0.531
Smoking	2.35 (0.75–7.37)	0.142	1.74 (0.59–5.14)	0.314
Initial NIHSS score	0.81 (0.74–0.89)	<0.001	0.82 (0.74–0.90)	<0.001
Use of intravenous tPA	2.72 (1.06–6.97)	0.037	2.66 (1.07–6.65)	0.036
ASPECTS	1.33 (1.10–1.61)	0.003		
Onset-to-recanalization time (per 30 min)	0.97 (0.93–1.00)	0.086	0.96 (0.93–1.00)	0.069
Hypoperfusion intensity ratio (per 0.1)	0.84 (0.68–1.03)	0.094	0.76 (0.62–0.92)	0.006

aOR, adjusted odds ratio; CI, confidence interval; NIHSS, National Institutes of Health Stroke Scale; tPA, tissue plasminogen activator; ASPECTS, Alberta Stroke Program Early Computed Tomography Score.

**Table 3:** Effects of the Alberta Stroke Program Early Computed Tomography Score (ASPECTS) and onset-to-recanalization time on a favorable outcome in the low hypoperfusion intensity ratio (HIR) group and the high HIR group.

Variables	Low HIR Group (*n* = 91)	*p*-Value	High HIR Group (*n* = 71)	*p*-Value
	aOR (95% CI)		aOR (95% CI)	
Age	0.96 (0.92–1.01)	0.137		
Diabetes			0.26 (0.07–1.01)	0.051
Smoking	2.70 (0.42–17.6)	0.298		
Initial NIHSS score	0.82 (0.73–0.93)	0.002	0.80 (0.68–0.95)	0.010
Use of intravenous tPA	2.34 (0.67–8.16)	0.182	4.27 (0.76–23.9)	0.098
ASPECTS	1.20 (0.92–1.56)	0.178	1.49 (1.09–2.03)	0.012
Onset-to-recanalization time (per 30 min)	0.97 (0.94–1.01)	0.194	0.92 (0.79–1.07)	0.283

aOR, adjusted odds ratio; CI, confidence interval; NIHSS, National Institutes of Health Stroke Scale; tPA, tissue plasminogen activator; ASPECTS, Alberta Stroke Program Early Computed Tomography Score.

## Data Availability

The data presented in this study are available on request from the corresponding author.
